# Osteogenesis Potential of Polymethylmethacrylate–Hydroxyapatite and Stem Cells from Human Exfoliated Deciduous Teeth as Alveolar Bone Graft: An *In* *Silico* Study

**DOI:** 10.1055/s-0044-1801280

**Published:** 2025-03-12

**Authors:** Tania Saskianti, Michelle Angelina, Ardianti Maartina Dewi, Yulanda Antonius, Amelinda Nabila Zahri, Rini Devijanti Ridwan, Masami Kanawa, Takeshi Kawamoto, Kotaro Tanimoto, Katsumi Fujimoto

**Affiliations:** 1 Department of Pediatric Dentistry, Faculty of Dental Medicine, Universitas Airlangga, Surabaya, Indonesia; 2 Biomaterial and Tissue Engineering Research Center, Universitas Airlangga, Surabaya, Indonesia; 3 Faculty of Biotechnology, University of Surabaya, Surabaya, Indonesia; 4 Department of Oral Biology, Faculty of Dental Medicine, Universitas Airlangga, Surabaya, Indonesia; 5 Research and Development Division, Department of Biomedical Science, Natural Science Center for Basic Research and Development, Hiroshima University, Hiroshima, Japan; 6 Writing Center, Hiroshima University, Hiroshima, Japan; 7 Department of Orthodontics and Craniofacial Developmental Biology, Graduate School of Biomedical and Health Sciences, Hiroshima University, Hiroshima, Japan; 8 Department of Molecular Biology and Biochemistry, Graduate School of Biomedical and Health Sciences, Hiroshima University, Hiroshima, Japan

**Keywords:** *insilico*, human and health, polymethylmethacrylate, hydroxyapatite, stem cells from human exfoliated deciduous teeth, alveolar bone defect therapy

## Abstract

**Objective:**

The goal is to analyze the osteogenesis potential of polymethylmethacrylate (PMMA)–hydroxyapatite (HA) and stem cells from human exfoliated deciduous teeth (SHED) as a biomaterial candidate for alveolar bone defect therapy through a bioinformatic approach within an *in silico* study.

**Materials and Methods:**

Three-dimensional (3D) ligand structures consisting of HA, PMMA, and target proteins of SHED were obtained from the PubChem database. STITCH was used for SHED target protein analysis, STRING was utilized for analysis and visualization of protein pathways related to osteogenesis, PASS Online was employed to predict biological functions supporting osteogenesis potential, PyRx 0.8 was used for molecular docking analysis, and PyMol was utilized to visualize the 3D structures resulting from the molecular docking analysis.

**Results:**

PMMA ligand was found to support osteogenesis through several biological functions, while interaction of HA ligand with matrix metalloproteinase (MMP) 20, DSPP, IBSP, SPP1, CD44, and MMP7 protein was revealed to play a role specifically in extracellular matrix organization. The interaction of all these proteins played a role in various pathways of osteogenesis. Toxicity level predictions of PMMA and HA were at class V and class III, respectively, which means that both ligands were shown to be neither hepatotoxic, carcinogenic, immunotoxic, nor cytotoxic. However, the ligand of PMMA had a lower binding affinity to SHED's protein (MMP7, MMP20, CD44, BMP7, and COL1A1) than the control ligand.

**Conclusion:**

The interaction between HA–PMMA ligands and several SHED proteins showed biological process and osteogenesis pathways supporting the osteogenesis potential of PMMA–HA and SHED for alveolar bone defect therapy.

## Introduction

Defects of the alveolar bone can be caused by several reasons, such as congenital anomalies, 1 drug consumption, 2 inflammation, 3 trauma, malignancy, 4 and dental surgical interventions, including periodontitis. 5 6 Alveolar bone defects caused by periodontal disease in children aged 2 to 11 years have a significant prevalence of 8.88%. 7 Furthermore, 75% of all cleft lip and palate variations are also accompanied by alveolar bone abnormalities, with an incidence rate of 1.596 patients, indicating that there are still many cases of alveolar bone defects occurring in children. 8 9 The gold standard graft for reconstructing alveolar bone defects is autogenous bone due to its osteogenic, osteoconductive, and osteoinductive properties. 6 However, the use of autogenous bone as grafts is not ideal for children, as it is invasive and may raise postoperative pain, infection, paresthesia, and the formation of scar tissues. 10 Therefore, alternative biomaterials capable of inducing bone formation still need to be developed.

Excellent osteoconductivity/inductivity properties are crucial for the long-term success of bone graft rehabilitation. The use of hydroxyapatite (HA) can influence osteoblast adhesion and proliferation and also increase osteoinduction. However, HA exhibits limited mechanical characteristics, including reduced tensile and compressive strength; therefore, supplementary reinforcing materials are commonly incorporated. 6 Polymethylmethacrylate (PMMA) is a polymer commonly used in the medical field as an adhesive in orthopaedics or as a bone filler. 11 PMMA does not have osteogenic or osteoconductive properties, but it is flexible and has good mechanical strength. The osteogenic and osteoconductive properties of HA can strengthen the function of PMMA, thereby producing a biomaterial that is easily manipulated and can induce osteogenic properties with decent mechanical strength. 12 As a candidate bone graft material, PMMA–HA has been reported to demonstrate antibacterial and antifungal abilities, thus reducing the risk of postoperative complications stemming from bacterial or fungal infections. 13 Of several ratios that were chemically characterized, 20:80 PMMA–HA was considered the optimal proportion for synthetic graft material. 14

Human exfoliated deciduous teeth (SHED) are an ideal source to be utilized in bone regeneration due to their positive results in osteogenic differentiation. 15 The combination of PMMA–HA with SHED is proposed as a bone graft material candidate based on the advantages of each ingredient that complement one another. However, for clinical use, the initial data on the interaction between PMMA–HA and SHED related to osteogenesis is crucial. Thus, this study aims to analyze the osteogenesis potential of PMMA–HA and SHED as a biomaterial candidate for alveolar bone defect therapy through a bioinformatic approach within an *in silico* study. This study was done simultaneously with *in vitro* research, which tested physical, 14 antibacterial, and antifungal properties, 13 as well as the cytotoxicity 16 of PMMA–HA; however, our *in vivo* research (animal experiment) analyzing the effect of PMMA–HA on osteogenesis has not been published yet.

## Materials and Methods

### Ligand Data Retrieval

A total of two ligand molecules, namely, PMMA (CID 6658) and HA (CID 18986957), were obtained from the PubChem database ( http://pubchem.ncbi.nlm.nih.gov ). The data collected consisted of the canonical Simplified Molecular Input Line Entry System (SMILES) notation and the three-dimensional (3D) structure of each molecule (in SDF format). Three proteins in SHED that are related to the process of osteogenesis consisting of matrix metalloproteinase (MMP) 7, MMP20, and CD44, as well as two bone biomarker proteins consisting of BMP7 and COL1A1, were collected from the PDB database.

### Ligand Biological Function Prediction

The biological functions of each ligand were predicted using the PASS Online Webserver ( https://www.way2drug.com/passonline/ ). The probability to be active value (Pa) was used as a parameter in various biological processes related to osteogenesis.

### Protein–Protein Interaction Network Analysis

Ligands were further analyzed to determine the protein–protein interaction network (PPIN) or protein pathways. The analysis was performed using the STITCH database ( http://stitch.embl.de/ ) and the STRING database ( https://string-db.org/ ). The false discovery rate (FDR) value was used as data validity.

### Visualization of PPIN

The protein network, which is composed of protein–protein targets from ligands known to contribute to the osteogenesis process, was visualized using the STRING database ( https://string-db.org/ ).

### Ligand Toxicity Prediction

The toxicity level of each ligand was predicted using the ProTox 3.0 Webserver ( https://comptox.charite.de/protox3/ ). Amount of dose (mg/kg) and toxicity class were selected as specific parameters. The accuracy data parameter was used to measure the data validity. Moreover, various toxicity models were predicted for further analysis.

### Molecular Docking Analysis and Molecular Visualization

Molecular docking analysis was performed using the PyRx 0.8 software. The strength interaction between the ligand and target protein was determined by the binding affinity score (kcal/mol). The protein's native ligand, a synthetic inhibitor, was used as a control compound. The 3D structure of ligand–protein complex as a result of molecular docking analysis was then visualized using the PyMol software.

## Results

### Ligand Data Retrieval

The SMILES notation and the 3D structure of two ligand molecules—PMMA (PubChem ID 665) and HA (PubChem ID 14781)—were obtained ( Table 1 ). Three proteins in SHED that are related to the process of osteogenesis consisting of MMP7 (PDB ID 7WXX), MMP20 (PDB ID 2JSD), and CD44 (PDB ID 4PZ3), as well as two bone biomarker proteins consisting of BMP7 and COL1A1, were collected from the PDB database.

**Table 1:** Data collection of PMMA and HA

Ligands	ID number	SMILES
PMMA	6658	CC(=C)C(=O)OC
HA	14781	[OH-].[O-]P(=O)([O-])[O-].[O-]P(=O)([O-])[O-].[O-]P(=O)([O-])[O-].[Ca + 2].[Ca + 2].[Ca + 2].[Ca + 2].[Ca + 2]

Abbreviations: HA, hydroxyapatite; PMMA, polymethylmethacrylate; SMILES, Simplified Molecular Input Line Entry System.

### Biological Functions of PMMA that Support Osteogenesis

Several biological functions of PMMA were predicted, including growth hormone agonist, antibacterial, immunosuppressant, MMP9 expression inhibitor, and skin irritation inactive with a Pa value ranging from 0.255 to 0.945, consecutively ( Table 2 ). A Pa value close to 1 indicated that the predicted biological function is highly valid, as it is based on *in vivo* research data.

**Table 2:** Biological function of PMMA prediction

Pa	Pi	Biological function
0.945	0.003	Skin irritation inactive
0.521	0.025	MMP9 expression inhibitor
0.337	0.093	Immunosuppressant
0.308	0.057	Antibacterial
0.255	0.071	Growth hormone agonist

Abbreviations: MMP, matrix metalloproteinase; Pa, probability activity; Pi, probability inactivity; PMMA, polymethylmethacrylate.

### PMMA–HA and SHED Protein Interaction Network

**Fig. 1:**
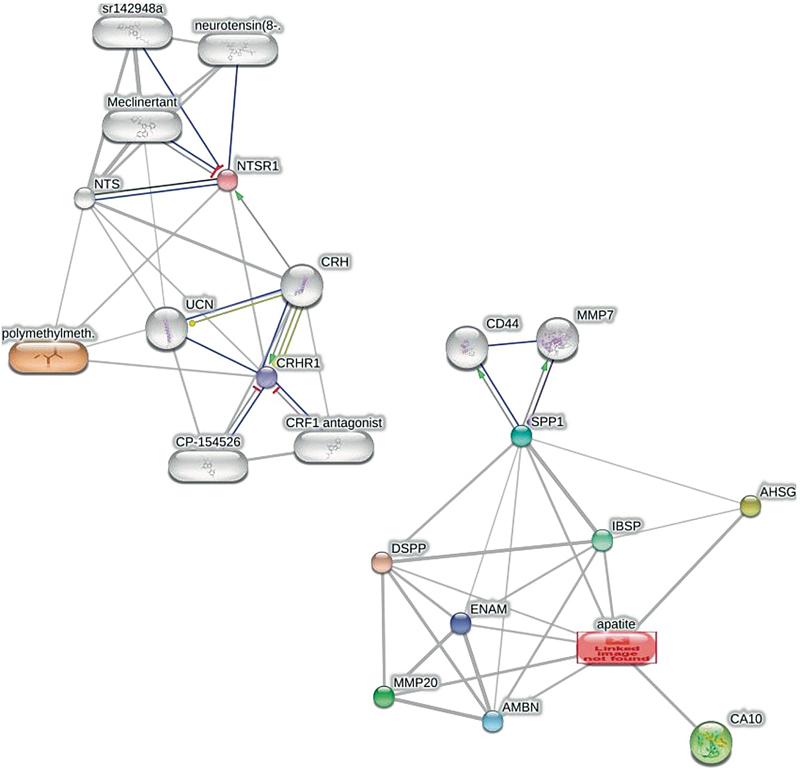
Visualization of polymethylmethacrylate (PMMA)–hydroxyapatite (HA0 protein–protein interaction.

Visualization showed that HA can interact directly (colored nodes) with several SHED proteins, namely, CA10, AMBN, ENAM, MMP20, IBSP, AHSG, DSPP, and SPP1. HA had an indirect interaction (uncolored nodes) with CD44 and MMP7. Meanwhile, PMMA directly interacted with CRHR1 and NTSR1 ( Fig. 1 ).

### Interaction of HA with SHED Proteins Related to the Extracellular Matrix Organization Pathway

**Fig. 2:**
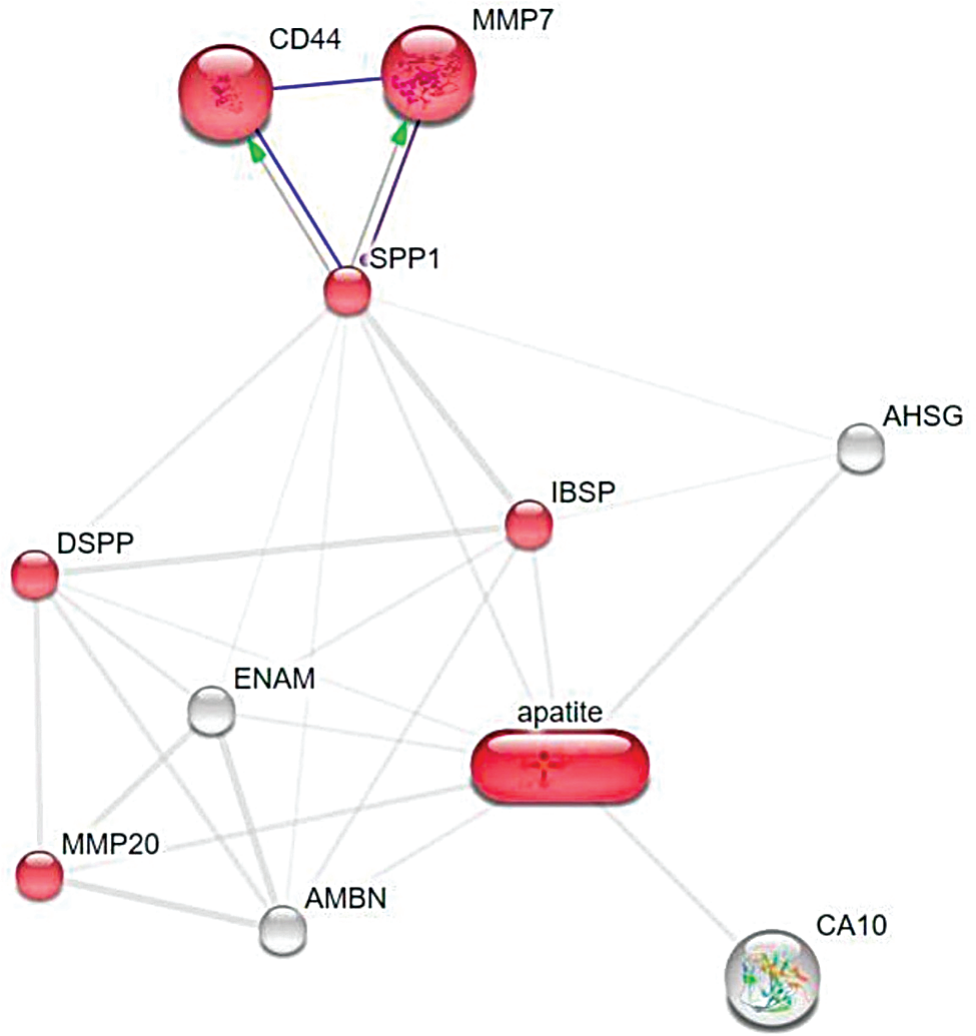
Ligand–protein interaction in the extracellular matrix (ECM) organization pathway.

HA interacted with MMP20, DSPP, IBSP, and SPP1, playing a role in the extracellular matrix (ECM) organization pathway. SPP1 had an activation function toward CD44 and MMP7, with a score of 0.800. This shows a high activation potential ( Fig. 2 ).

### Osteogenic Biological Processes of PMMA–HA and SHED

**Fig. 3:**
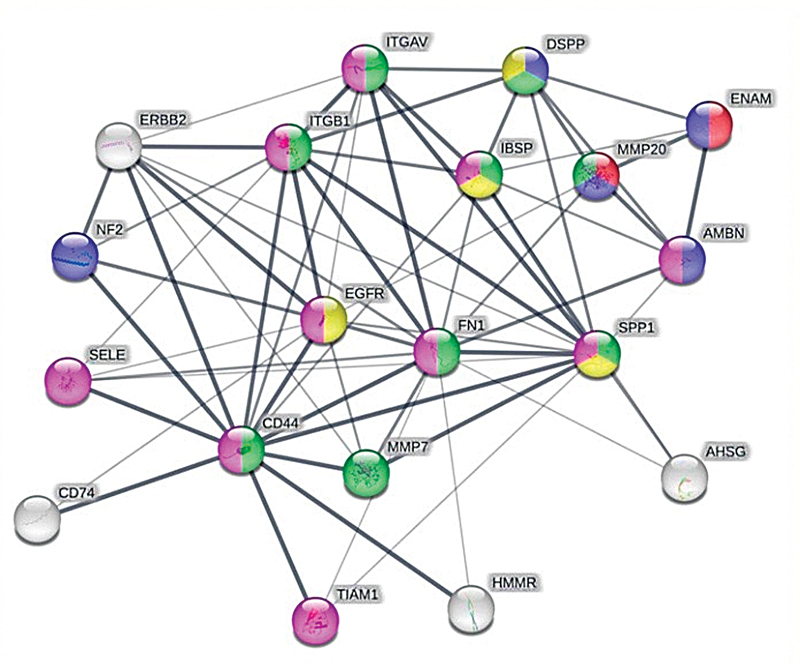
Protein–protein interaction in the osteogenesis pathway.

SHED proteins that can interact with HA were involved in several osteogenesis pathways. Each protein was then visualized ( Fig. 3 ) and analyzed further to determine the mechanism of interaction among proteins or protein pathways ( Table 3 ). Each pathway is shown by a different color: regulation of enamel mineralization (red), odontogenesis of dentin-containing tooth (blue), ECM organization (green), ossification (yellow), and cell adhesion (magenta).

**Table 3:** Predicted protein–protein interactions in the osteogenesis pathway

Pathway ID	Biological process	Protein	FDR
GO:0070173	Regulation of enamel mineralization (red)	**MMP20** , ENAM	0.0044
GO:0042475	Odontogenesis of dentin-containing tooth (blue)	**MMP20** , ENAM, AMBN, DSPP, NF2	3.44e-05
GO:0030198	ECM organization (green)	**MMP20,** DSPP, ITGAV, IBSP, ITGB1, SPP1, FN1, **MMP7, CD44**	1.58e-07
GO:0001503	Ossification (yellow)	SPP1, EGFR, IBSP, DSPP	0.0171
GO:0007155	Cell adhesion (magenta)	TIAM1, **CD44,** SELE, FN1, EGFR, SPP1, ITGB1, ITGAV, IBSP, AMBN	1.57e-05

Abbreviations: AMBN, ameloblastin; CD44, acidic cell surface adhesion protein; DSPP, dentin sialophosphoprotein; EGFR, epidermal growth factor receptor; ENAM, enamelogenin; FDR, false discovery rate; FN1, fibronectin 1; IBSP, integrin-binding sialoprotein; ITGAV, integrin subunit α V; ITGB1, integrin subunit β 1; MMP7, matrix metalloproteinase 7; MMP20, matrix metalloproteinase 20; NF2, neurofibromatosis type 2; SELE, selectin E; SPP1, secreted phosphoprotein; TIAM1, T cell lymphoma invasion and metastasis 1.

### Ligand Toxicity Prediction

The toxicity class of PMMA was determined to be class V, meaning that it may be harmful if more than 3,625 mg/kg was swallowed. Meanwhile, the toxicity class of HA was class III, indicating that this ligand would be toxic if more than 3,920 mg/kg was swallowed. Both ligands were shown to be neither hepatotoxic, carcinogenic, immunotoxic, nor cytotoxic ( Table 4 ).

**Table 4:** Ligand toxicity level prediction

Ligands	LD50 (mg/kg)	Toxicity class	Accuracy	Toxicity model			
				Hepatotoxicity	Carcinogenicity	Immunotoxicity	Cytotoxicity
PMMA	3625	V	100%	Inactive	Inactive	Inactive	Inactive
HA	3920	III	70.97%	Inactive	Inactive	Inactive	Inactive

Abbreviations: HA, hydroxyapatite; LD50, lethal dose 50/median lethal dose; PMMA, polymethylmethacrylate.

### Binding Affinity of PMMA and Target Proteins

**Fig. 4:**
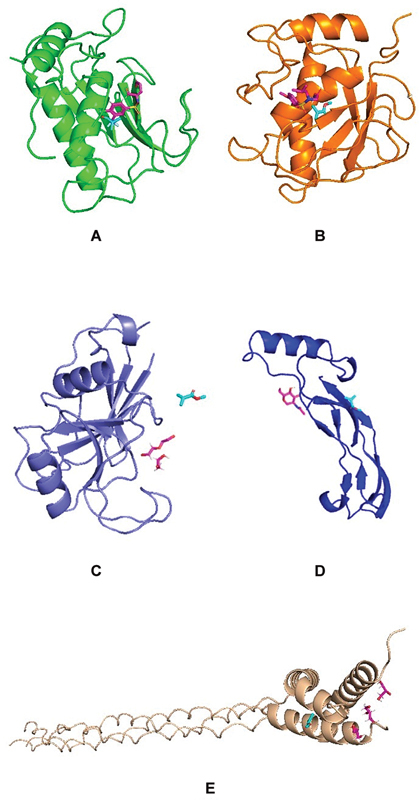
Molecular visualization of polymethylmethacrylate (PMMA) and matrix metalloproteinase (MMP) 7 (green) with its native ligand ( **A** ); MMP20 (orange) with its native ligand ( **B** ); CD44 (purple) with PMMA with its native ligand ( **C** ); BMP7 (dark blue) with its native ligand ( **D** ); and COL1A1 (wheat) with its native ligand ( **E** ).

Visualization of complex ligand and target protein showed that each ligand had the same position point as the native ligand, which indicated that those ligands bind in the same active site location ( Fig. 4 ). However, the binding affinity of PMMA and target SHED proteins—MMP7, MMP20, CD44 BMP7, and COL1A1—was low compared with their native ligands ( Table 5 ).

**Table 5:** Binding affinity prediction between PMMA and native ligand with target proteins

Target proteins	Grid center coordinates	Binding affinity (kcal/mol)	
		PMMA	Native ligand (control)
MMP7	X: −28.615 Y: −21.488 Z: −6.916	−3.4	−6.2
MMP20	X: 8.147 Y: −5.319 Z: 7.806	−4.8	−5.4
CD44	X: 11.605 Y: 5.782 Z: 15.368	−3.5	−5.3
BMP7	X: 1.706 Y: 79.270 Z: 16.120	−3.6	−4.9
COL1A1	X: 55.947 Y: 32.406 Z: 23.488	−3.2	−6.6

Abbreviations: BMP7, bone morphogenetic 7; CD44, acidic cell surface adhesion protein; COL1A1, collagen type I α 1; MMP7, matrix metalloproteinase 7; MMP20, matrix metalloproteinase 20; PMMA, polymethylmethacrylate.

## Discussion

Interest in synthetic bone graft alternatives has grown in recent decades. One of the ideal benefits of a synthetic bone graft is that it can stimulate bone; therefore, the creation of synthetic materials with osteoconductive and osteoinductive qualities capable of eliminating the requirement for harvesting has emerged as a significant goal of synthetic bone transplant research. In this study, we used a bioinformatic technique to test the osteogenesis potential of PMMA–HA and SHED via an *in silico* study. SMILE notations of the PMMA and HA were collected from the PubChem database ( Table 1 ). Furthermore, the 3D structure of each compound of interest was collected for further analysis.

PMMA and HA ligands were further analyzed to determine the biological function of each ligand related to the osteogenesis process in SHED. This prediction result was based on the probability activity (Pa) and probability inactivity (Pi) values. A Pa value close to 1 indicates that the biological function has been tested based on the validity of the study up to the *in vivo* scale. PMMA ligand binding had biological functions that supported osteogenesis potential but did not play a direct role in the process. This result is in line with the research by Oryan et al, who concluded that PMMA has low bioactive properties and does not significantly increase bone regeneration. 17 This research indicated that PMMA ligands can support osteogenesis potential through skin irritation inactive processes, MMP9 expression inhibitors, immunosuppressants, and growth hormone agonists ( Table 2 ). In this research, it was shown that PMMA had biological functions that support the osteogenesis process. The highest biological activity potency was skin irritation inactive function. PMMA was often used in the treatment of craniofacial tissue defects because it does not contain potentially harmful subunits, such as bisphenol A found in polycarbonate. 18

PMMA and HA had interaction network toward various protein nodes, which are depicted by the edges. In brief, those interaction networks are related to osteogenesis-related proteins. Several proteins are related to SHED, including AMBN, ENAM, DSPP, IBSP, and SPP1 ( Fig. 1 ). Moreover, further interaction network among protein–protein related to PMMA and HA showed that various pathways are involved in the osteogenesis pathway. It consisted of regulation of enamel mineralization, odontogenesis of dentin tooth, ECM organization, ossification, and cell adhesion. Various pathways revealed various linked proteins. However, there are also several proteins that have contributed to other pathways ( Fig. 3 , Table 3 ). These proteins are regarded as essential protein, which may impact various mechanism pathways. It could possibly serve as a target for the therapeutic development.

The osteogenesis pathway demonstrated by the interaction of the HA ligand with the SHED protein is mainly due to its role in the ECM organization process ( Fig. 2 ). The ECM organization process has a low proportion of false positives, as indicated by its low FDR value. ECM organization is a cellular mechanism where the assembly and disassembly of several ECM components occur (GO:0030198). This is closely related to the bone formation process, where bone ECM can dynamically interact with osteoblast cells to regulate new bone formation. 19 Several proteins, such as MMP20, DSPP, IBSP, SPP1, CD44, and MMP7, are some of the SHED proteins involved in this process. ECM is a noncellular 3D structure secreted by cells into the extracellular space. Bone ECM consists of several proteins and polysaccharides and contains inorganic and organic compounds. In bone, ECM plays a role in regulating cell adhesion, proliferation, response to growth factors, differentiation, and characteristics of mature bone. Bone ECM can induce the production of new bone by osteoprogenitor cells, such as mesenchymal cells, osteoblasts, and osteocytes. 20 21

Another mechanism involved in the osteogenesis pathway of HA ligand interactions with SHED proteins is the process of odontogenesis of dentin-containing teeth, the specific result of which is the development of dentin-containing teeth (GO:0042475). Teeth are mineralized organs that include enamel, dentin, and cementum. AMBN and ENAM proteins are needed in the process of tooth development (odontogenesis) through enamel mineralization. MMP20 also plays an important role in the amelogenesis process in tooth development by degrading amelogenin and ameloblastin. Bone is also a mineralized tissue, so it can be concluded that apart from the process of tooth development, these three proteins play a role in the development and differentiation of bone as a mineralized tissue. 22 23 24

Ossification is a bone formation process involving osteoblasts, osteoclasts, and osteocytes (GO:0001503). Ossification is a biological process that supports the osteogenesis pathway in the interaction of the HA ligand with the SHED protein in terms of the FDR value ( Table 3 ). DSPP, IBSP, SPP1, and AHSG play a role in regulating the activity of osteoblasts and osteoclasts. The bone matrix will surround the osteoblast cells, and then the matrix will be converted into mature bone cells, namely, osteocytes. 25

The PMMA and HA ligands were then analyzed for toxicity to predict the safe-use level of the ligand. This is an important step, as it indicates the appropriate level of the ligand being utilized. Both PMMA and HA were relatively safe to be used as bone graft material, as they were shown to be neither hepatotoxic, carcinogenic, immunotoxic, nor cytotoxic ( Table 4 ). The most widely used synthetic material for bone grafts is HA, as it closely resembles the mineral component of the human bone. When tested with human dental pulp stem cells, HA exhibits lower cytotoxicity compared with synthetic and bovine-derived materials as some synthetic materials showed higher concentrations of toxic elements like tungsten. 26 A previous research using nano-HA also confirmed its biocompatibility with no significant adverse effects. 27 Another widely used biomaterial for bone grafts is PMMA, which has also been proved safe as it was found to be nontoxic to mouse fibroblast cell lines. 28 Our previous research confirms the safety issue of PMMA–HA. 16 We did an *in vitro* toxicity test of PMMA–HA simultaneously with this *in* *silico* research. Mixed PMMA–HA was shown to be nontoxic for SHED and osteoblasts *in vitro* . 16 This shows that our *in silico* prediction results are in line with the *in vitro* experimental results.

Five proteins that were related to the osteogenesis pathway, such as MMP20, BMP7, MMP7, CD44, and COL1A1, were selected as target proteins for simulation of protein–ligand binding interaction. The bioinformatics approach was conducted to analyze its interaction between ligand PMMA and each protein for comprehensive analysis by using molecular docking. PMMA is the only ligand employed since its 3D structure could be completely generated. 29

In brief, the molecular docking analysis aimed to identify the binding strength among ligand PMMHA to the active site of various proteins related to osteogenesis pathway. The binding strength is expressed as a binding affinity score in negative value. Lower binding affinity score or more negative the binding affinity score determined, the stronger the interaction between the ligand and the target protein. 30 The binding affinity score of PMMA is consistently compared with the native ligand from each target protein. The native ligand is served as a control. Results depicted that PMMA had relatively comparable binding affinity score as compared with native ligand of each target protein. However, the interaction of PMMA toward MMP20 and BMP7 is relatively similar as control ( Table 5 ). In brief, in the treatment of alveolar defects, there was no standardized biomaterial of choice that could be used as a control. Therefore, a native ligand was used as a control. Research conducted by Prahasanti et al showed similar results; specifically, HA–PMMA and BMP7 had binding affinity values that were similar as the results in this study. 31 There was no previous research regarding the binding affinity scores of PMMA toward CD44, COL1A1, MMP7, and MMP20. 32

## Conclusion

According to the research, it was concluded that there was an interaction between PMMA–HA ligands and SHED proteins. The HA ligand interaction with SHED protein is related to osteogenesis, specifically in ECM organization, while PMMA ligand interaction does not play a direct role in osteogenesis. PMMA ligands are predicted to have several biological processes that can support osteogenesis. Both PMMA and HA are relatively safe to be used as bone graft material. This is the initial condition for proposing an alternate material to mend the alveolar bone defect. *In* *silico* research comparing the PMMA–HA molecular complex to PMMA and HA in their individual conditions is needed. Furthermore, *in vivo* animal research is required to demonstrate the effectiveness of PMMA–HA in the alveolar defect model.
